# *In Vitro* Activity of Selected West African Medicinal Plants against *Mycobacterium ulcerans* Disease

**DOI:** 10.3390/molecules21040445

**Published:** 2016-04-13

**Authors:** Patrick Valere Tsouh Fokou, Abena Adomah Kissi-Twum, Dorothy Yeboah-Manu, Regina Appiah-Opong, Phyllis Addo, Lauve Rachel Tchokouaha Yamthe, Alvine Ngoutane Mfopa, Fabrice Fekam Boyom, Alexander Kwadwo Nyarko

**Affiliations:** 1 Department of Clinical Pathology, Noguchi Memorial Institute for Medical Research, College of Health Sciences, University of Ghana, Accra LG 581, Ghana; abena.adoma@live.com (A.A.K.-T.); RAppiah-Opong@noguchi.ug.edu.gh (R.A.-O.); ANyarko@noguchi.ug.edu.gh (A.K.N.); 2 Antimicrobial Agents Unit, LPMPS, Department of Biochemistry, Faculty of Science, University of Yaoundé 1, Yaoundé 812, Cameroon; yamthe_lauve@yahoo.fr (L.R.T.Y.); ngmfalvine@yahoo.fr (A.N.M.); fabrice.boyom@fulbrightmail.org (F.F.B.); 3 Department of Bacteriology, Noguchi Memorial Institute for Medical Research, University of Ghana, Accra LG 581, Ghana; DYeboah-Manu@noguchi.ug.edu.gh; 4 Department of Animal Experimentation, Noguchi Memorial Institute for Medical Research, University of Ghana, Accra LG 581, Ghana; PAddo@noguchi.ug.edu.gh; 5 Institute of Medical Research and Medicinal Plants Studies (IMPM), Yaoundé 6163, Cameroon; 6 Department of Pharmacology and Toxicology, School of Pharmacy, University of Ghana, Accra LG 43, Ghana

**Keywords:** medicinal plants, Buruli ulcer, cytotoxicity, *Mycobacterium ulcerans*

## Abstract

Buruli ulcer (BU) is the third most prevalent mycobacteriosis, after tuberculosis and leprosy. The currently recommended combination of rifampicin-streptomycin suffers from side effects and poor compliance, which leads to reliance on local herbal remedies. The objective of this study was to investigate the antimycobacterial properties and toxicity of selected medicinal plants. Sixty-five extracts from 27 plant species were screened against *Mycobacterium ulcerans* and *Mycobacterium smegmatis*, using the Resazurin Microtiter Assay (REMA). The cytotoxicity of promising extracts was assayed on normal Chang liver cells by an MTT assay. Twenty five extracts showed activity with minimal inhibitory concentration (MIC) values ranging from 16 µg/mL to 250 µg/mL against *M. smegmatis*, while 17 showed activity against *M. ulcerans* with MIC values ranging from 125 µg/mL to 250 µg/mL. In most of the cases, plant extracts with antimycobacterial activity showed no cytotoxicity on normal human liver cells. Exception were *Carica papaya*, *Cleistopholis patens*, and *Polyalthia suaveolens* with 50% cell cytotoxic concentrations (CC_50_) ranging from 3.8 to 223 µg/mL. These preliminary results support the use of some West African plants in the treatment of Buruli ulcer. Meanwhile, further studies are required to isolate and characterize the active ingredients in the extracts.

## 1. Introduction

Medicinal plants are used around the world as either prescriptions and/or over-the-counter therapeutics for the prevention and treatment of diseases [1]. In Africa, the vast majority (70%–80%) of people consult traditional medical practitioners for their healthcare needs [2], including the management of Buruli ulcer disease [3].

Buruli ulcer, also known as Bairnsdale ulcer, Daintree ulcer, or Mossman ulcer is a necrotizing infection of the skin and subcutaneous tissue [4]. Its pathogenesis has been associated with mycolactone, a lipidic exotoxin encoded by *M. ulcerans*’ specific giant plasmid that has cytotoxic and immunosuppressive properties [5]. It is the third most common mycobacterial infection in the world, after tuberculosis and leprosy, and one of the most neglected but treatable tropical diseases [6]. Amongst health priorities in the world, the control of infectious diseases including Buruli ulcer ranks high. The successful implementation of rifampicin-streptomycin combination therapy as the first-line treatment option for Buruli ulcer disease and early case detection have significantly reduced the number of surgical interventions associated with BU, long stays in health facilities and relapse rates. However, rifampicin-streptomycin combination therapy suffers from challenges due to: (1) potential toxicity causing hearing impairment, especially in children who are the most affected in Africa, and 8 weeks of pain associated with parenteral administration of streptomycin; (2) drug-drug interactions caused by rifampicin; and (3) indirect cost to families due to long stays in hospital associated with in-patient treatment and/or daily transport costs for outpatient case management. Due to these drawbacks, poor compliance is not uncommon, which is a predisposing factor for the occurrence of drug resistant bacterial strains. Consequently, a compelling need to search for new compounds with antimycobacterial activity for improved treatment of Buruli ulcer is critical in resource-poor settings of Africa where the disease burden is the highest. A vibrant drug discovery pipeline is therefore needed to help ensure the availability of new products that will reduce morbidity resulting from this infection.

Fortunately, Africa is endowed with vital resources, that is diversified and rich flora on which the population depend as sources of remedies for various ailments [7,8]. This traditional medicine is more affordable and accessible compared to the standard or modern medicine in-use to manage neglected tropical diseases such as Buruli ulcer. In fact, the majority of the populations affected by Buruli ulcer live in remote rural areas with poor health care systems, life-threatening poverty and bad road networks to carry patients to health facilities. This study was designed to screen extracts from some West African herbs for antimycobacterial activity, using the causative agent of Buruli ulcer, *M. ulcerans*, and the less pathogenic but fast growing *M. smegmatis*.

## 2. Results and Discussion

### 2.1. Selection of Plants and Extracts Preparation

In our search for natural products with antimycobacterial activity, 27 native plants of Cameroon and Ghana belonging to 12 plant families were selected based on their use to treat Buruli ulcer, wounds, and related symptoms. A total of 65 crude extracts were prepared from the plants using hexane, dichloromethane, ethanol, methanol, and hydroethanol and screened against *M. smegmatis* and *M. ulcerans*. The plants parts investigated in this work, the extraction yields as well as the voucher specimen numbers of plants are given in Table 1.

### 2.2. Antimycobacterial Activity of Plants Extracts

The *in vitro* antimycobacterial activity of the plants extracts against *M. smegmatis* and *M. ulcerans* were measured by the REMA assay. The results obtained as shown in Table 2 indicated that of the 65 tested extracts, 25 exerted activity with MIC values ranging from 16 µg/mL to 250 µg/mL against *M. smegmatis* while 17 extracts showed anti-*M. ulcerans* activity, with MIC values ranging from 125 to 250 µg/mL.

Globally 16 out 17 active extracts from 10 plant species, *viz. Alstonia boonei*, *Annona reticulata*, *Annona senegalensis*, *Bridelia ferruginea*, *Cariya papaya*, *Eucaluptus globulus*, *Plyalthia suaveolens*, *Sorindeia juglandifolia*, *Spathodea campanulata*, and *Zanthoxylum zanthoxyloides* showed activity with an MIC of 250 µg/mL, and one extract from *Cleistopholis patens* at MIC equal to 125 µg/mL against *M. ulcerans* (Table 2). The published criterion [9] used in considering a plant extract promising for further study against *M. ulcerans* is an MIC value lower or equal to 250 µg/mL.

Of the 25 extracts that showed activity against *M. smegmatis*, nine had MICs of 250 µg/mL, another nine had MIC values of 125 µg/mL, three had MIC values of 63 µg/mL, another three had MIC values of 31 µg/mL, and one had an MIC value of 16 µg/mL (Table 2). Further investigations are ongoing to scrutinize their antimycobacterial properties. Overall, as shown in Table 2, 44% (11/25) of extracts that were active against *M. smegmatis* also inhibited *M. ulcerans*, suggesting that *M*. *smegmatis* might be a good model for identifying active ingredients against *M*. *ulcerans.*

For the antimycobacterial assay on *M. smegmatis*, Annonacee species, namely *Annona muricata*, *Annona reticulata, Annona senegalensis, Artabotrys rufus, Annickia chlorantha, Cleistopholis patens*, and *Polyalthia suaveolens* displayed the highest antimycobacterial activity, with MICs ranging from 16–250 µg/mL (Table 2). Addo *et al.* [10] and Donkeng *et al.* [11] previously demonstrated that *Annickia chlorantha*, *Polyalthia suaveolens*, *Phyllanthus fraternus*, *Sorindeia juglandifolia*, and *Zanthoxylum zanthoxyloides* have antimycobacterial activity against *M. bovis* BCG, *M. tuberculosis*, and *M. ulcerans*. These authors also showed that the stem bark extracts of *Annickia chlorantha* and *Polyalthia suaveolens* exerted antimycobacterial activity against *M. smegmatis* with respective MIC values of 250 and 6250 µg/mL [10], but improved activity was obtained from our work at 125 µg/mL and 16–250 µg/mL (Table 2), respectively for the trunk and stem extracts of *Annickia chlorantha*. Moreover, the previous investigators showed that extracts from the fruit, leaf, pulp, pericarp, root, seed, and twig of *Annona muricata* demonstrated no activity. In contrast, the stem and root extracts of this plant showed activity in the present study with MIC values ranging between 125 and 250 µg/mL (Table 2). Previous studies on Annonaceous plants indicated the presence of nitrogenous compounds such as cleistopholine in *Cleistopholis patens* and bidebiline E in *Polyalthia cerasoides* that possess antimycobacterial properties [12]. The stem bark extract of *Azadirachta indica* moderately inhibited *M. smegmatis* at 250 µg/mL, supporting the antimycobacterial activity of azadiradione isolated from this plant [13]. Besides, reports also indicate the traditional use of *Azadirachta indica* for the treatment of leprosy [14]. *M. smegmatis* was also inhibited by the leaf extract of *Ricinus communis* at 125 µg/mL, corroborating the activity of the leaf acetone extract of this plant reported previously to have an MIC value as low as 20 μg/mL [15].

Plant extracts that showed activity against *M. ulcerans*, were from different families, *viz.* Annonaceae, Apocynaceae, Bignoniaceae, Caricaceae, Compositae, Euphorbiaceae, Myrtaceae, Phyllanthaceae, and Rutaceae. The strongest anti-*M. ulcerans* activity was observed for the extract of *Cleistopholis patens* stem bark with an MIC value of 125 µg/mL (Table 2). A study conducted in the Amansie West District, which is the most endemic area in Ghana indicated that *Cleistopholis patens* is one of the indigenous plants used for the treatment of Buruli ulcer and the positive responses alluded to by previous users suggest its effectiveness [7]. Our findings also corroborate the usefulness of *Cleistopholis patens* in BU management. Equally, our recent review reported that *Alstonia boonei*, *Bridelia ferruginea*, *Carica papaya*, *Spathodea campanulata*, and *Zanthoxylum zanthoxyloides* are used indigenously to treat Buruli ulcer [3]. It is also worth noting that, for the first time, this study has reported the anti-*M. ulcerans* activity *in vitro* of *Carica papaya* and *Cleistopholis patens*. Previously, aqueous extracts of *Alstonia boonei*, *Bridelia ferruginea*, *Spathodea campanulata*, and *Zanthoxylum zanthoxyloides* were evaluated for antimycobacterial activity against *M. ulcerans* [10] and presented MIC values ranging from 13 to 40 µg/mL that are lower than those obtained in our study. A tentative explanation of this difference in MIC values might be differences in the assays used in the two studies as well as extraction procedures used. Addo and collaborators used the proportion method and aqueous extraction, while we used the resazurin microtiter assay and 70% ethanolic extract. On the other hand, previous investigators found *Jathropha curcas* to be active but *Bridellia ferruginea* and *Vernonia amygdalina* inactive [9] respectively which are in contrast with the results of this study. This could be due to differences in the geographical location where plants were collected, which is known to have an impact on the composition of secondary metabolites of the plants.

The other plants that showed anti-*M. ulcerans* activity are *Annona reticulata*, *Annona senegalensis*, *Eucalyptus globulus*, *Polyalthia suaveolens*, and *Sorindeia juglandifolia*, which are traditionally used in the treatment of skin diseases including cancer, furuncles, abscesses, pain, wound healing and poorly healing ulcers, edema and swollen glands [16,17,18,19,20]. Their activities support their potential to be used in the treatment of Buruli ulcer. Also, while the crude ethanolic extract of *Polyalthia suaveolens* stembark was previously shown to inhibit *M. ulcerans* with moderate MIC value of 3125 µg/mL [11], our study demonstrated that the trunk extract has a higher activity with an MIC value of 250 µg/mL. This suggests that the trunk of *Polyalthia suaveolens* might contain more promising active components compared to the stembark. Furthermore, the ethanolic fruit extract of *Sorindeia juglandifolia* also showed activity against *M. ulcerans* in our study (MIC = 250 µg/mL; Table 2), corroborating the findings from previous work that indicated an MIC of 63 µg/mL. This observed activity is likely to be due to the presence of the compound 2,3,6-trihydroxy methyl benzoate that was shown to inhibit the growth of *M. ulcerans* strain 1615 with an MIC value of 63 µg/mL [11].

### 2.3. Cell-Based Toxicity Results

The extracts that showed promise were further assessed for cytotoxicity against Chang liver cells (normal human liver cells). The results, shown in Table 2, indicate that the majority of active extracts showed no cytotoxicity at concentrations up to 250 µg/mL. However, four extracts from *Carica papaya* (leaf and stem bark), *Cleistopholis patens* (stembark), and *Polyalthia suaveolens* (trunk) showed deleterious effects on cells, with CC_50_ values ranging from 3.8 to 223 µg/mL, the most cytotoxic being the ethanolic extracts of *Carica papaya* leaf (CC_50_ = 3.8 µg/mL) and stembark (CC_50_ = 54 µg/mL). Overall, many of the cytotoxic extracts displayed CC_50_ values many orders of magnitude above the 30 µg/mL considered as limit value to qualify a plant extract as cytotoxic according to the American National Cancer Institute [21]. These results suggest awareness that plant based remedies should be taken with caution by those who use them.

### 2.4. Preliminary Phytochemical Analysis

Qualitative preliminary phytochemical analysis was performed on the promising extracts to detect the phytoconstituents that might be responsible for the observed antimycobacterial activity (Table 3, Figure S1). The types of secondary metabolites varied according to the plant and organ tested, and the solvent used for extraction. Overall, the screening of extracts revealed the presence of phenols, tannins, flavonoids, saponins, alkaloids, anthraquinones, glycosides, and triterpenes (Table 3), which are known to have antimycobacterial properties [22]. The phytoconstituents present in extracts that reacted with 7.5% (*v*/*v*) sulfuric acid spray reagent were also separated by thin layer chromatography using a chloroform/ethyl acetate/formic acid (5:4:1) [CEF] solvent system (Figure S1). This preliminary screening may give the first hints of the presence of metabolites of the mentioned classes but these results must be corroborated by more specific phytochemical analyses. More than 17 compounds varying in polarity were separated. The established chemical profile of crude extracts will provide guidance for further investigations of the active ingredients for their pharmacological properties.

In conclusion, the data obtained in this work demonstrate that some extracts from the West African plants that we studied have activities against *M. ulcerans* and *M. smegmatis*. Further studies are required to isolate and characterize the bioactive secondary metabolites that are responsible for the observed anti-*M. ulcerans* properties to support the search for new plant-derived drugs against Buruli ulcer. In addition, prospective studies on the promising extracts will consolidate their potential formulation as phytomedicines to control BU in remote West African settings and elsewhere.

## 3. Materials and Methods

### 3.1. Plant Collection

Twenty seven plants were selected based on the available literature about medicinal plants used in West Africa for the treatment of Buruli ulcer, wounds and related symptoms. Selected plants from Ghana and Cameroon were collected and identified with the help of taxonomists either in Ghana or Cameroon. Voucher specimens of all plants selected for the study were deposited in herbaria at the Centre for Plant Medicine Research (CPMR), Mampong-Akuapem, Ghana and Cameroon National Herbarium, respectively.

### 3.2. Preparation of Plant Extracts

The fresh plants materials were cut into small pieces, dried under shade, and then ground to a fine powder using an electric mill. Samples from Cameroon were first of all subjected to maceration in ethanol, then liquid-liquid extraction. Liquid-liquid extraction is a powerful sample preparation method, based on the selective partitioning of analytes between two immiscible liquid phases. It is simple, fast, and efficient in the extraction of non-volatile constituents. Analytes dissolved in water were partitioned by means of the immiscible organic solvent, dichloromethane, and then the dried dichloromethane extract was partitioned between methanol and hexane [23]. Afforded extracts were shipped to Noguchi Memorial Institute for Medical Research, Accra, Ghana for bioassays. Plants materials from Ghana were prepared and extracted using hydroethanol (H_2_O–ethanol 70:30), a suitable system to extract the majority of non-volatile constituents. Rotary evaporation and freeze-drying were used to remove organic solvents and water, respectively. The evaporated and freeze-dried extracts were stored at −20 °C until use. Extracts were individually weighed and the percentage yield of extraction was calculated relative to the weight of the starting dried plant material (Table 1). Stock solutions for screening were prepared in 100% DMSO at 25 mg/mL.

### 3.3. In vitro Antimycobacterial Assays

#### 3.3.1. Propagation of Mycobacterial Strains

Our in-house reference *M. ulcerans* NM209 strain stored at −80 °C was thawed and subcultured onto Middlebrook 7H11 agar (Difco, Sparks, MD, USA) containing 10% oleic acid-dextrose-catalase complex (OADC) and incubated at 31 °C until confluent growth was observed. Sterility was checked by subculturing on blood agar and microscopy after Ziehl-Neelsen staining. *M. smegmatis* strain mc^2^ 155 from ETH Zurich was grown at 37 °C on 7H11 agar (Difco).

#### 3.3.2. Drug Susceptibility Assays: Determination of MIC

Plant extracts were tested for anti-mycobacterial activity using the Resazurin Microtiter Assay [8] in clear-bottomed, 96-well microplates. Stock solutions of the tested extracts were prepared in high-grade dimethyl sulfoxide (DMSO) (Sigma-Aldrich, Steinheim, Germany) at a concentration of 25 mg/mL and diluted further with Middlebrook 7H9 broth (Difco) for the assays at concentrations ≤250 µg/mL. Considering earlier reported effects of DMSO [9] on *M. ulcerans*, we performed a set of control experiments that showed that 1% DMSO did not have any inhibitory effect on the growth of either *M. ulcerans* NM209 or *M. smegmatis* mc^2^ 155 in three independent duplicates. However, the highest concentration of DMSO in reaction wells for the screening of our plant extracts was 0.98%.

Mycobacterial inoculum was prepared by emulsifying a loop full of growing mycobacteria in sterile Middlebrook 7H9 broth supplemented with 0.2% glycerol (Sigma-Aldrich, Steinheim, Germany) (7H9-GC) and adjusted to 1 × 10^6^ cells/mL. All perimeter wells of the plate were filled with 200 µL of sterile water to prevent evaporation of the content of inner wells. The test wells contained 7H9-G medium containing 10% OADC (100 μL) and respective concentrations of testing drugs, which were serially diluted to final concentrations of 16 to 250 μg/mL, and 100 μL of the mycobacterial inoculum.

Rifampicin and streptomycin were used as positive controls, while culture medium alone and culture medium with mycobacterium were the blank and negative control, respectively. The plates were incubated at 31 °C for 15 days for *M. ulcerans* and 48 h for *M. smegmatis* at 37 °C. Bacterial viability was thereafter determined by adding 20 μL of resazurin (Acros Organics, Morris, NJ, USA) at 0.01% (*w*/*v*), and the plates further incubated at 31 °C for 48 h for *M. ulcerans* and overnight for *M. smegmatis* at 37 °C. Dye colour changing from blue to red indicated bacterial growth. The results were considered if and only if the negative control wells became red after addition of the resazurin. MIC values were defined as the lowest concentration of extract that prevented a color change of resazurin.

### 3.4. Cytotoxicity Assay

The cytotoxicity of the extracts was tested against the Human Chang liver cell using the MTT assay. The Chang liver cell line was obtained from Dr. Takuhiro Uto, Department of Pharmacognosy, Faculty of Pharmaceutical Sciences, Nagasaki International University (Nagasaki, Japan). Cells were maintained in RPMI 1640 (Sigma, Ayrshire, UK). From sub-confluent cultures in 75 cm^2^ culture flasks, they were trypsinized, counted, suspended in RPMI containing 10% Fetal bovine serum and 1% penicillin-streptomycin-glutamine (Sigma) and then seeded into triplicate wells of a 96-well plate (100 µL per well) at concentrations of 1 × 10^5^ cells/mL and incubated at standard culture conditions of 5% CO_2_ in air at 37 °C. Cells were allowed to attach overnight and then treated with 10 µL per well of 2-fold serially diluted individual plant extracts (15.125–250 µg/mL) in the culture medium and incubated for 48 h. After incubation, 20 µL of 2.5 mg/mL MTT (Sigma) solution were added and plates were incubated for additional 4 h. Acidified isopropanol (150 μL) with Triton X-100 was added to the plates to solubilize the formazan formed. Following an overnight incubation, the optical densities were read using an Infinite 200 PRO spectrophotometer (Tecan, Grodig, Austria) at a wavelength of 570 nm. Curcumin was used as positive control in all assays. The viability of cells was evaluated based on a comparison with untreated cells. Data were normalized to percent control activity and CC_50_ values representing the sample’s concentration required to inhibit 50% of cell proliferation were calculated using GraphPad Prism 5.0 software (GraphPad Software Inc., San Diego, CA, USA) with data fitted by nonlinear regression.

### 3.5. Phytochemical Analysis of Active Extracts

The phytochemical screening of promising extracts was carried out to detect the extracts constituents that might sustain the biological activity. To achieve this, phenols, tannins, flavonoids, saponins, alkaloids, anthraquinones, anthocyanins, glycosides, and triterpenes were screened using previously described procedures [24,25]. In addition, the extracts were also chromatographically analysed by thin layer chromatography (TLC) (Silica gel F254, Merck, Darmstadt, Germany). The mobile phases consisted of chloroform/ethyl acetate/formic acid (5:4:1) [CEF]. The separated components on TLC plates were examined under UV light at 365 nm and further sprayed with 7.5% (*v*/*v*) sulfuric acid and heated at 100 °C for optimal colour development.

## Figures and Tables

**Table 1:** Brief overview of plants used for the *in vitro* anti-mycobacterial studies.

Plant Family	Plant Species	Country of Collection	Voucher Specimen Number	Plant Part Tested (x) and Extraction Yield (%)
Anacardiaceae	*Mangifera indica* L.	Ghana	CSRPM/010914	R(he) (6.2); L(he) (10.9)
	*Sorindeia juglandifolia* (A.Rich.) Planch. ex Oliv.	Cameroon	9176 SRF/Cam	F(e) (13); T(e) (1.4); L(e) (5.4)
Annonaceae	*Annickia chlorantha* (Oliv.) Setten & Maas	Cameroon	32,065/SRF/Cam	SB(m) (1.2); SB(e) (2.9)
	*Annona muricata* L.	Cameroon	32,879/HNC	P(e) (5.7); S(m) (4.8); SB(e) (5.9); Sd (m) (3.5); R(m) (3.5); R(e) (6.2)
	*Annona reticulata* L.	Cameroon	66,886/HNC	F(e) (5.1); T(e) (5); R(e) (5.1); L(e) (10.4); SB(d) (3.7); T(d); SB(e) (6.02); L(he) (1.04); F(d) (9.9); R(d) (4.5)
	*Annona senegalensis* Pers.	Cameroon	40,060/HNC	S(e) (2.5); SB(d) (1.5); T(e) (1.8); S(d) (1.04); L(e) (11.6)
	*Artabotrys rufus* De Wild.	Cameroon	48,757/HNC	L(e) (5.0); S(e) (4.02)
	*Cleistopholis patens* (Benth.) Engl. & Diels	Cameroon	23,169/SRF/Cam	SB(d) (5.5); T(e) (7.5); L(d) (5.5); L(e) (10.5)
	*Polyalthia suaveolens* Engl. & Diels	Cameroon	1227/SRF/CAM	SB(e) (4); T(e) (2.0); Tr(d) (0.4); S(e) (3.7); Tr(e) (1.54); S(d) (0.5); SB(d) (2.9); T(d) (0.73)
Apocynaceae	*Alstonia boonei* De Wild.	Ghana	CSRPM/100714	L(he) (7.78)
	*Holarrhena floribunda* (G.Don) T. Durand & Schinz	Ghana	CSRPM/021214	SB(he) (6.93); L(he) (7.8)
Bignoniaceae	*Spathodea campanulata* P. Beauv.	Ghana	CSRPM/120714	R(he) (6.63)
Caricaceae	*Carica papaya* L.	Ghana	CSRPM/070714	L(he) (16.0); R(he) (5.06)
		Cameroon	18647/SFRCam	L(e) (15.1); SB(e) (5.54)
Compositae	*Ageratum conyzoides* (L.) L.	Ghana	CSRPM/090714	L(he) (10.23)
	*Chromolaena odorata* (L.) R.M.King & H.Rob.	Ghana	CSRPM/011214	L(he) (8.96)
	*Vernonia amygdalina* Delile	Ghana	CSRPM/060714	L(he) (7.7)
		Cameroon	42362/HNC	L(e) (6.0)
Euphorbiaceae	*Alchornea cordifolia* (Schumach. & Thonn.) Müll.Arg.	Ghana	CSRPM/110714	L(he) (10.2)
	*Jatropha curcas* L.	Ghana	CSRPM/030714	L(he) (13)
	*Ricinus communis* L.	Ghana	CSRPM/031214	L(he) (7.4)
Meliaceae	*Azadirachta indica* A.Juss.	Ghana	CSRPM/010714	SB(he) (15)
Myrtaceae	*Eucalyptus globulus* Labill.	Cameroon	4077/SRFC	L(e) (8.7)
Phyllanthaceae	*Bridelia ferruginea* Benth	Ghana	CSRPM/080714	L(he) (7.4); SB(he) (7)
	*Phyllanthus fraternus* G.L.Webster	Ghana	CSRPM/040714	WP(he) (40)
Rutaceae	*Zanthoxylum zanthoxyloides* (Lam.) Zepern. & Timler	Ghana	CSRPM/090714	R(he) (10.4)
Solanaceae	*Solanum erianthum* D. Don	Ghana	CSRPM/020914	L(he) (7.02); F(he) (9.5)
	*Solanum torvum* Sw.	Ghana	CSRPM/050714	L(he) (8.9)

L: leaf; SB: stem bark; R: root (wood + bark); S: stem (wood + bark); F: fruit; Sd: seed; P: Pericarp; T: twig; Tr: Trunk (stem − bark); WP: Whole plant. (x). Solvents used for extraction: h, hexane; he, 70% hydroethanolic solution; d, dichloromethane; e, ethanol; m, methanol.

**Table 2:** Antimycobacteral activity of the plant extracts.

Plant Species	Part	Extract Code	*MIC (µg/mL) on *M. smegmatis*	*MIC (µg/mL) on *M. ulcerans*	CC_50_ (µg/mL) Chang Liver Cell
*Mangifera indica* L.	Root	MIR (he)	>250	>250	ND
	Leaf	MIL (he)	250	>250	ND
*Sorindeia juglandifolia* (A.Rich.) Planch. ex Oliv.	Twig	SJT (e)	>250	>250	ND
	Leaf	SJL (e)	>250	>250	ND
	Fruit	SJF (e)	63	250	>250
*Annona muricata* L.	Pericarp	AMP (e)	>250	>250	ND
	Stem	AMS (m)	250	>250	ND
	Stem bark	AMSB (e)	>250	>250	ND
	Seed	AMSd (m)	>250	>250	ND
	Root	AMR (m)	250	>250	ND
	Root	AMR (e)	125	>250	ND
*Annona reticulata* L.	Fruit	ARF (e)	31	>250	>250
	Twig	ART (e)	>250	>250	
	Root	ARR (e)	250	250	>250
	Leaf	ARL (e)	>250	>250	ND
	Stem bark	ARSB (d)	63	>250	ND
	Twig	ART (d)	>250	>250	ND
	Stem bark	ARSB (e)	125	>250	ND
	Leaf	ARL (he)	>250	>250	ND
	Fruit	ARF (d)	31	250	>250
	Root	ARR (d)	125	250	>250
*Annona senegalensis* L.	Stem	ASS (e)	>250	>250	ND
	Stem bark	ASSB (d)	>250	>250	ND
	Twig	AST (e)	>100	>100	ND
	Stem	ASS (d)	31	250	ND
	Leaf	ASL (e)	>250	>250	ND
*Artabotrys rufus* De Wild.	Leaf	ArRL (d)	125	>125	ND
	Stem	ArRS (e)	250	>250	ND
*Cleistopholis patens* (Benth.) Engl. & Diels	Stem bark	CPSB (d)	125	125	20.8 ± 2
	Twig	CPT (e)	125	>250	ND
	Leaf	CPL (d)	ND	>250	ND
	Leaf	CPL (e)	>250	>250	ND
*Annickia chlorantha* (Oliv.) Setten & Maas syn	Stem bark	ACSB (m)	125	250	ND
	Stem bark	ACSB (e)	ND	>250	ND
*Polyalthia suaveolens* Engl. & Diels	Stem bark	PSSB (e)	>250	>250	ND
	Twig	PST (e)	250	>250	ND
	Trunk	PSTr (d)	>250	>250	ND
	Stem	PSS (e)	>250	>250	ND
	Trunk	PSTr (e)	>250	250	223 ± 2
	Stem	PSS (d)	16	250	>250
	Stem bark	PSSB (d)	>250	250	>250
	Twig	PST (d)	>250	>250	ND
*Alstonia boonei* De Wild.	Leaf	ABL (he)	>250	250	>250
*Holarrhena floribunda* (G.Don) T. Durand et Schinz	Stem bark	HFSB (he)	ND	>250	ND
	Leaf	HFL (he)	ND	>250	ND
*Spathodea campanulata* P. Beauv.	Root	SCR (he)	>250	250	>250
*Carica papaya* L.	Leaf	CPL (he)	>250	>250	ND
	Root	CPR (he)	>250	>250	ND
	Leaf	CPL (e)	>250	250	3.8 ± 1
	Stem bark	CPSB (e)	>250	250	54 ± 1
*Ageratum conyzoides* (L.) L.	Leaf	ACL (he)	>250	>250	ND
*Chromolaena odorata* (L.) R.M.King & H.Rob.	Leaf	COL (he)	ND	>250	ND
*Vernonia amygdalina* Delile	Leaf	VAL (he)	>250	>250	ND
	Leaf	VAL (e)	>250	250	ND
*Alchornea cordifolia* (Schumach. & Thonn.) Müll.Arg.	Leaf	ACL (he)	250	>250	ND
*Jatropha curcas* L.	Leaf	JCL (he)	>250	>250	ND
*Ricinus communis* L.	Leaf	RCL (he)	125	>250	ND
*Azadirachta indica* A.Juss.	Stem bark	AISB (he)	250	>250	ND
*Eucalyptus globulus* Labill.	Leaf	EGL (e)	125	250	>250
*Bridelia ferruginea* Benth	Leaf	BFL (he)	>250	>250	ND
	Stem bark	BFSB (he)	>250	250	ND
*Phyllanthus fraternus* G.L.Webster	Whole plant	PFWP (he)	250	>250	ND
*Zanthoxylum zanthoxyloides* (Lam.) Zepern. & Timler	Root	ZZR (he)	63	250	>250
*Solanum erianthum* D. Don	Leaf	SEL (he)	ND	>250	ND
	Fruit	SEF (he)	ND	>250	ND
*Solanum torvum* Sw.	Leaf	STL (he)	>250	>250	ND
Streptomycin		*/*	ND	1	/
Rifampicin		*/*	8	0.12	/
Curcumin		/	/	/	31 ± 2

(x). Solvents used for extraction: h, hexane; he, 70% hydroethanolic solution; d, dichloromethane; e, ethanol; m, methanol. Activity data presented are means of two replicates. ND: Not determined. *MICs were measured by the REMA assay.

**Table 3:** Qualitative phytochemical composition of the most promising extracts.

Plant Species	Part	Extract Code	Phenols	Tannins	Flavonoids	Saponines	Alkaloids	Anthraquinones	Anthocyanns	Glycosides	Triterpenes
*Sorindeia juglandifolia* (A.Rich.) Planch. ex Oliv.	Fruit	SJF (e)	+	+	+	+	-	+	-	+	+
*Annona muricata* L.	Stem	AMS (m)									
	Root	AMR (m)	+	+	+	-	+	-	-	+	+
	Root	AMR (e)	+	+	+	+	+	-	-	+	+
*Annona reticulata* L.	Fruit	ARF (e)	+	+	+-	-	+	+	-	+	+
	Root	ARR (e)	+	+	+	-	+	+	-	+	+
	Stem bark	ARSB (d)	+	+	+	-	+	+	-	+	+
	Stem bark	ARSB (e)	+	+	+	+	+	+	-	+	+
	Fruit	ARF (d)	+	+	+	-	+	+	-	+	+
	Root	ARR (d)	+	-	+	-	+	+	-	+	+
*Annona senegalensis* L.	Stem	ASS(d)	+	-	+	-	+	+	-	+	+
*Artabotrys rufus* De Wild.	Leaf	ArL (d)	+	-	+	+	+	-	-	+	+
	Stem	ArS (e)	+	+	+	+	+	-	-	+	+
*Cleistopholis patens* (Benth.) Engl. & Diels	Stem bark	CPSB (d)	+	-	+	+	+	-	+	+	+
	Twig	CPT (e)	+	-	+	+	+	-	-	+	+
*Annickia chlorantha* (Oliv.) Setten & Maas syn	Stem bark	ANSB (m)	+	-	-	+	+	-	-	+	+
*Polyalthia suaveolens* Engl. & Diels	Twig	PST (e)	+	+	-	+	-	ND	ND	-	-
	Trunk	PSTr (e)	+	-	+	+	+	ND	ND	-	-
	Stem	PSS(d)	+	+	+	+	+	ND	ND	+	+
*Alstonia boonei* De Wild.	Leaf	ABL (he)	+	+	+	+	+	-	-	-	-
*Spathodea campanulata* P. Beauv.	Root	SCR (he)	-	+	+	+	-	-	-	-	-
*Carica papaya* L.	Leaf	CPL (e)	-	+	-	+	+	ND	ND	+	+
	Stem bark	CPSB (e)	+	+	-	+	+	-	ND	+	-
*Vernonia amygdalina* Delile	Leaf	VAL (e)	+	+	+	+	+	ND	ND	+	+
*Eucalyptus globulus* Labill.	Leaf	EGL (e)	+	+	+	-	+	ND	ND	-	-
*Bridelia ferruginea* Benth	Stem bark	BFSB (he)	+	+	+	+	-	-	-	-	-
*Zanthoxylum zanthoxyloides* (Lam.) Zepern. & Timler	Root	ZZR (he)	+	+	+	+	+	-	-	-	-

Note: + indicates presence of metabolite type; – indicates absence of metabolite type.

## Supplementary Materials

Supplementary materials can be accessed at: http://www.mdpi.com/1420-3049/21/4/445/s1.

## Abbreviations

The following abbreviations are used in this manuscript:

BU	Buruli ulcer
MIC	Minimum Inhibitory Concentration
CC50	Sample’s concentrations required to inhibit 50% of cell proliferation
CPMR	Centre for Plant Medicine Research
