# Supplementary material for: In Vitro Activity of Selected West African Medicinal Plants against Mycobacterium ulcerans Disease
Source: Molecules. 2016 Apr 13;21(4):445. doi: 10.3390/molecules21040445 (PMC6273889; doi:10.3390/molecules21040445)
Supplement: Supplementary file 1 [file molecules-21-00445-s001.pdf]

Supplementary Materials: *In Vitro* Activity of Selected West African Medicinal Plants against *Mycobacterium ulcerans* Disease

Patrick Valere Tsouh Fokou, Abena Adomah Kissi-Twum, Dorothy Yeboah-Manu, Regina Appiah-Opong, Phyllis Addo, Lauve Rachel Tchokouaha Yamthe, Alvine Ngoutane Mfopa, Fabrice Fekam Boyom and Alexander Kwadwo Nyarko

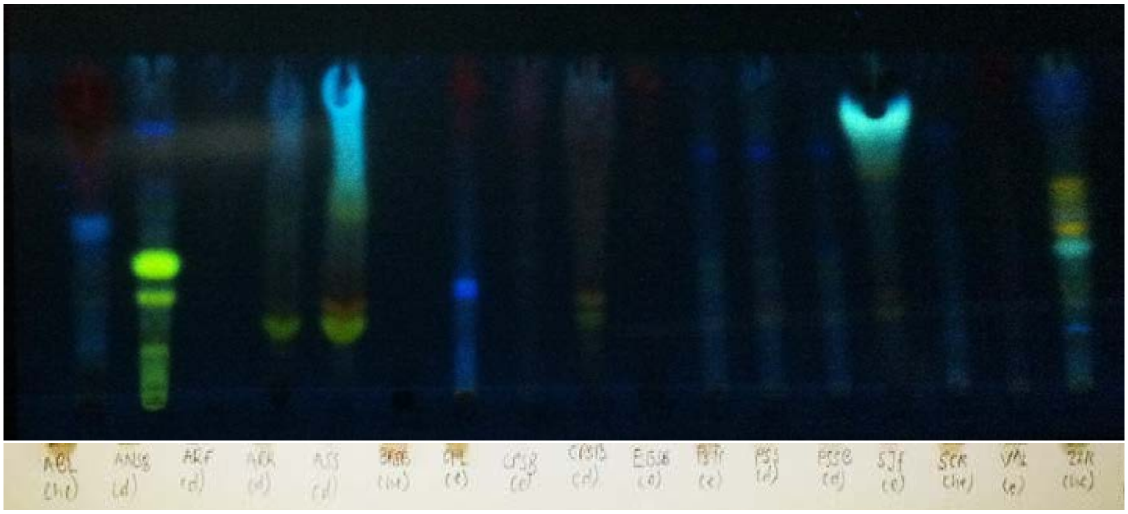

A

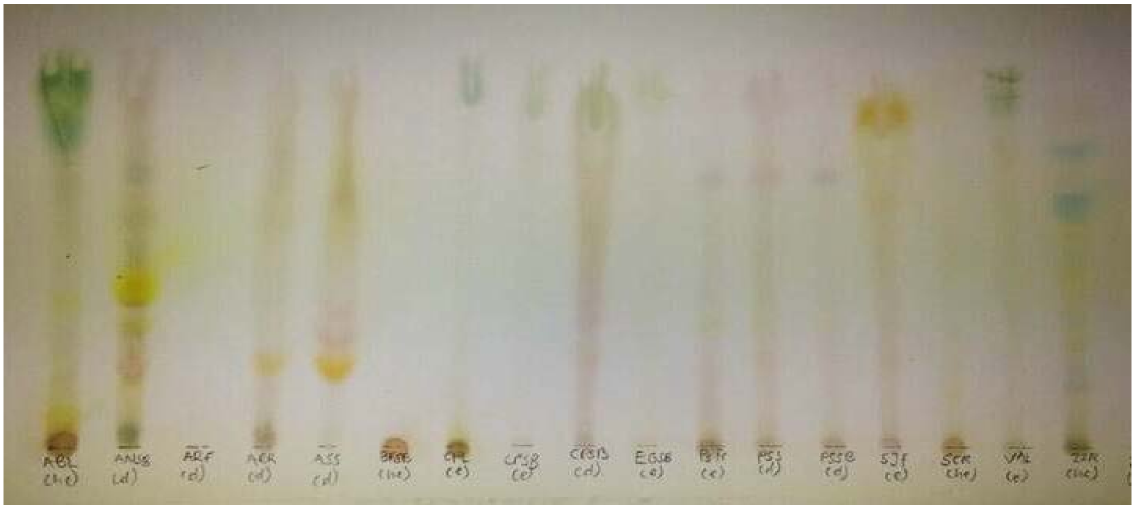

B

Figure S1. (**A**) Under the UV lamp at 365 nm; (**B**) After spraying with 7.5 % (*v*/*v*) sulfuric acid and heated at 100 °C. TLC plates developed in CEF system and sprayed with 7.5 % (*v*/*v*) sulfuric acid showing varied chemical constituents of the seventeen selected extracts. KEY: ABL(he), *Alstonia boonei* leaves; ANSB(d), *Annickia chloranta* stem bark; ARF(d) *Annona reticulate* fruit, ARR(d), *Annona reticulate* root; ASS(d), *Annona senegalensis* stem; BFSB(he), *Bridelia ferruginea* Stem bark; CPL(e), *Carica papaya* leaves; CPSB(e), *Carica papaya* stem bark; CPSB(d), *Cleistopholis patens* Stem bark; EGSB(e), *Eucaluptus globulus* leaves; PSTr(e), *Polyalthia suaveolens* trunk; PSS(d), *Polyalthia suaveolens* stem, PSSB(d); *Polyalthia suaveolens* stem bark; SJF(e), *Sorindeia juglandifolia* fruit; SCR(he); *Spathodea campanulata* roots; VAL(e), *Vernonia amygdalina* leaves; ZZR(he), *Zanthoxylan zanthoxyloides* roots. A: Under the UV lamp at 365 nm; B: After spraying with 7.5 % (*v*/*v*) sulfuric acid and heated at 100 °C.
